# Heavy Metals Contamination of Table Salt Consumed in Iran

**Published:** 2010

**Authors:** Abdol Majid Cheraghali, Farzad Kobarfard, Noroldin Faeizy

**Affiliations:** a *Department of Toxicology and Pharmacology and Chemical Injuries Research Center, University of Baqiyatallah Medical Sciences, Tehran, Iran.*; b *Department of Medicinal Chemistry, Faculty of Pharmacy, Shaheed Beheshti University of Medical Sciences, Tehran, Iran.*

**Keywords:** Table salt, Rock salt, Heavy metal contamination, Iran

## Abstract

Lead, cadmium, mercury and arsenic are the most important heavy metals which may cause health risks following consumption of contaminated foods. Table salt is one the mostly used food additive with unique place in food consumption. Although purified table salt is expected to have lower level of contamination, some Iranians still prefer to use rock salt. Use of rock salt for food purposes has been banned by Iranian health authorities. In this study, heavy metal contamination of table salt consumed in Iran has been investigated. One hundred samples of rock and refined table salts were analyzed using atomic absorption spectrophotometeric methods for the presence of toxic heavy metals. The mean concentration of tested tracer metals including Cd, Pb, Hg and As was 0.024, 0.438, 0.021 and 0.094 μg/g, respectively. The concentrations of tested heavy metals were well below the maximum levels set by Codex. However, no statistically significant difference was found between contamination of rock salt and refined salt to heavy metals.

## Introduction

Although food-borne illnesses caused by consumption of food commodities contaminated with microorganisms and/or their toxins is the major public health risk, health hazard from contamination of foods with other toxins including fungal toxins and heavy metals could also create acute poisoning as well as long-term health problems. Despite outstanding improvement in providing healthy foods worldwide in recent decades, incidence of food contamination is still a valid concern which raises questions about their human health and economic consequences. Although it is expected that contamination of staple foods play major role in intoxication of consumers, contamination of food additives may also contribute to this phenomenon. In fact, since most of the producers and national regulators may overlook possible contamination of food additives, their role may be underestimated.

Lead (Pb), cadmium (Cd), mercury (Hg) and arsenic (As) are the most important heavy metals which may cause health risks from consumption of contaminated foods. Heavy metals have been in use in human societies in many different areas for thousands of years. Although adverse health effects of heavy metals have been known for a long time, exposure to heavy metals continues and in some countries is even increasing. Unfortunately foods and food containers is one of the major routes of heavy metal contamination in general population. 

Table salt is one the mostly used food additive with unique place in food consumption. Salt (sodium chloride) is an essential additive which routinely added to majorities of foods not only for improving taste but also as a preservative to many canned, salted and pickled or fresh foods. The harvest of salt from the surface of the salt lakes dates back to at least 6000 BC, making it one of the oldest food additives in human history. Refined salt, which is most widely used presently, is mainly sodium chloride. Every year, several hundreds of million tones of salt is produced worldwide. However, food grade salt accounts for only a small part of salt production in industrialized countries although worldwide food uses account for 17.5% of salt production. 

In spite of considerable variation, daily intake of salt for many consumers is substantial. Therefore, due to the daily consumption of table salt, any contamination in salt even in low level could create health risks to the consumers. Recently, incidence of heavy metal contamination in table salt has been investigated worldwide (1-5).

In order to improve iodine daily intake, Iran health authorities have encouraged use of crystallized and fortified table salt since past decades (6). Today, most refined salt is prepared from rock salt which is mined either conventionally or through the injection of water. Raw salt may be later refined through purification and recrystallization. In the latter process, a brine solution is treated with chemicals that precipitate most impurities (largely magnesium and calcium salts). Multiple stages of evaporation are then used to collect pure sodium chloride crystals. Although it is expected that purified table salt has lower level of contaminations, for cultural and economical reasons some Iranian still prefer to use rock salt. Rock salt or halite is a type of unpurified food salt directly obtained from salt mines and are presented to the consumers in different size and weights. Although direct human use of rock salt has been discouraged by the health authorities, its presence in the market indicates that this type of salt is still being used by the consumers. In this study, heavy metals contamination of table salt consumed in Iran both as rock salt and refined salt has been investigated.

## Experimental

**Materials**

Authentic sodium chloride standard (Merck, Germany) was used for preparation of calibration curve. Ammonium pyrolidine dithiocarbamate (APDC) and 4-methyl-2-pentanone (MIBK) and all reagents used in this study were of analytical grade. All solutions were prepared using double-deionized water. 

**Methods**

*Sampling*

Thirty refined and prepacked salt samples were purchased directly from the shops around the city of Tehran. In fact, at least one sample of refined salt was collected from every registered producer of refined salt in the country. Seventy samples of rock salts from different known mines of rock salt were collected either from restaurants (which are the main user of this type of salt) or vendors around the city. Twenty grams of each sample was used for analysis.

*Heavy metals measurements*

Since direct determination of heavy metals using flame atomic absorption spectroscopy (FAAS) in concentrated aqueous solutions of salt samples was not possible, a reported extraction method (7) was used for analysis of salt samples. Briefly, 20 g of salt sample was dissolved and diluted in 100 mL of double-distilled purified water in a 250 mL polyethylene flask. After adjusting pH to 4.4-4.8 using acetic acid-sodium acetate buffer, 5 mL of APDC and 10 mL of MIBK were added. Following five minutes of intense shaking of the mixture, organic phase was separated and its absorbance was measured using FAAS. A Perkin-Elmer 1100B model flame atomic absorption spectrometer equipped with deuterium lamp background correction was used for determination of heavy metals. 

A hydrogenation atomic absorption spectrometry method was used for As measurement. In this method, after adding 1 mL of nitric acid to 1 g of sample for digestion, HCl and NaBH_4 _was added to the mixture. Trapped ions were measured using a Varian Spect AA200 instrument. Hg content of the sample was measured using cold vapor atomic absorption spectrophotometery by a BUCK scientific 400A atomic absorption spectrophotometer. 

A separate calibration curve was established for each metal using standard solution. Unpaired t-test was used for statistical analysis of the results and P < 0.05 was considered as significant differences.

## Results and Discussion

**Figure 1:**
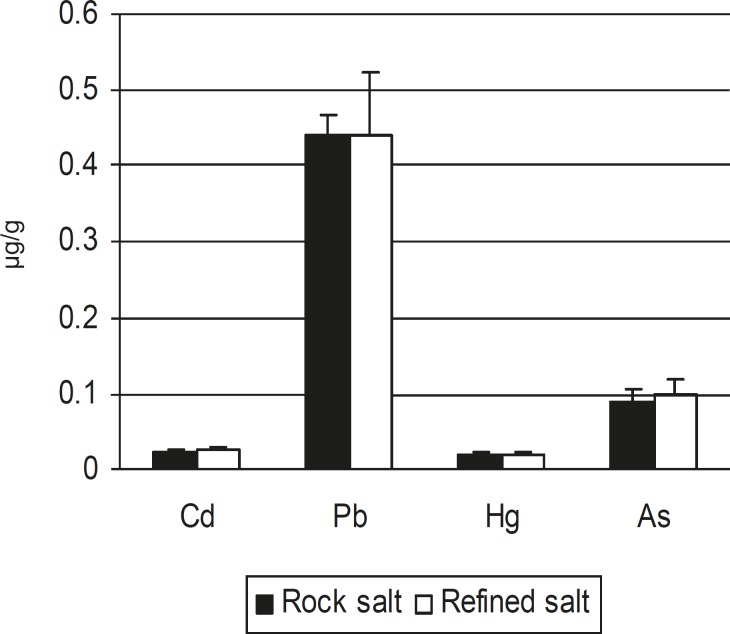
Comparison of heavy metal contamination of rock salt and refined salt consumed in Iran.

Results of samples analysis are summarized in Table 1. The mean concentration of tested tracer metals including Cd, Pb, Hg and As was 0.024, 0.438, 0.021 and 0.094 μg/g, respectively. Figure 1 shows comparative analysis of heavy metal contamination of rock salt and refined table salt. No statistically significant difference was found between these two groups (P > 0.05).

Salt is the most used food additive worldwide. Therefore, any contamination of table salt could be considered as a health hazard to the consumers. Since most of salt used around the globe comes from mines, it is expected that heavy metal contamination might be a concern for table salt. Due to known health hazard of toxic heavy metals to the consumers, contamination of food stuff to these metals should be avoided (8). The strong and consistent correlation reported between the intake of salt and salted food and the incidence of stomach cancer and other pre-cancerous lesions could be attributed to the possible contamination of salt with heavy metals especially arsenic (9, 10). Non-human use of salt either as an industrial agent, de icing agent or as an additive to cattle feed also might contribute to human intoxication. Any heavy metal contamination of salt is being used for non-food purposes may ultimately enter into human food chain. It has been reported that the salt used for road maintenance in winter ends up in the stream waters which may be used by human (11). Some investigators examined the presence of heavy metal contamination in mineral salts mixture commercially available as supplements of cattle feed. They reported substantially high concentration of lead and cadmium in these mixtures (12). It is expected that these contamination may finally end up in human food chain. 

In Iran, a national program of salt fortification with iodine has commenced since decades ago in order to prevent iodine-deficiency-related disease, and therefore use of refined and fortified table salt is strongly encouraged (6). Iran ministry of health also discourages use of rock and unrefined salt as food salt. However, due to some economical and cultural reasons still some people prefer to use rock salt. This practice is more common in restaurants. The present investigation has examined the presence of some important toxic heavy metals including Cd, Pb, Hg and As in different types of table salt consumed in Iran. As it is summarized in Table 1, concentration of tested toxic heavy metals in food salt consumed in Iran is well below established maximum limits for presence of toxic metals in table salt by Codex. According to Codex legislation, the maximum tolerated amounts of heavy metals in salt are 0.5 μg/g of As, 2 μg/g of Pb, 0.5 μg/g of Cd and 0.1 μg/g of Hg (13). The mean concentration of heavy metals including Cd, Pb, Hg and As found in table salt in Iran was 0.024, 0.438, 0.021 and 0.094 μg/g, respectively.

**Table 1:** The mean concentration of heavy metals in table salts consumed in Iran in comparison with Codex maximum limit

**Tracer**	**Mean ± SD (μg/g)**	**Codex maximum limit (μg/g)**
Cd	0.024±0.002	0.2
Pb	0.438±0.021	1.0
Hg	0.021±0.001	0.05
As	0.094±0.013	0.5

Recently, the heavy metal contents of refined and unrefined table salts from Turkey, Egypt and Greece have been studied (1). According to the reported data, the concentration of Pb in table salt was between 0.54-1.64 μg/g. The Cd level in these samples was below 0.3 μg/g. Dim et al. have found a 200 times higher concentration of Pb in local cooking salt comparing with other salts consumed in Nigeria (14). In a separate study Cd levels of table salts used in Nigeria reported as high as 4.5 μg/g (4). Concentrations of Pb and Cd in table salts consumed in Brazil reported to be in the range of 0.03-0.1 μg/g and 0.01-0.03 μg/g, respectively (5). It seems that Pb and Cd contents of table salts consumed in Iran are more or less similar to the values reported from other countries. However, Cd, Pb, Hg and As concentrations in table salts consumed in Iran are well below the maximum limits set by Codex (Table 1). 

In this investigation, toxic heavy metals content of rock salt was also compared with those of refined salt. As it can be seen in Figure 1, there was no significant difference between these two types of salt from the heavy metal contents point of view. 

In conclusion, evaluation of both rock salt and refined salt consumed in Iran for the presence of toxic heavy metals showed that the concentration of these tracer metals in table salt is well below the maximum levels set by Codex. Although it is expected that rock salts might have other impurities and/or insoluble substances, they do not impose more health hazard to the consumers due to the presence of higher concentrations of toxic heavy metals. Considering higher cost of refined salts for places with high consumption of salt e.g. restaurants or dormitories, use of rock salt may not be considered as breach of food safety as it is now the case from the Iranian health authorities point of view. 
